# Eliminating viral hepatitis C in Belgium: the micro-elimination approach

**DOI:** 10.1186/s12879-020-4898-y

**Published:** 2020-02-27

**Authors:** Dana Busschots, Samira Toghanian, Rob Bielen, Stina Salomonsson, Özgür M. Koc, Greet Hendrickx, Michel Jadoul, Frederik Nevens, Etienne Sokal, Christian Brixko, Kathelijne Peerlinck, Ludwig Apers, Geert Robaeys, Jeffrey V. Lazarus

**Affiliations:** 1 Faculty of Health and Life Sciences, Hasselt University, Diepenbeek, Belgium; 2 Department of Gastroenterology and Hepatology, Ziekenhuis Oost-Limburg, Genk, Belgium; 3 MSD, Centre of Observational Real-world Evidence (CORE), Stockholm, Sweden; 4 Medical Microbiology, School of NUTRIM, Maastricht University Medical Centre, Maastricht, the Netherlands; 5 Viral Hepatitis Prevention Board, Centre for the Evaluation of Vaccination, Vaccine & Infectious Diseases Institute, University of Antwerp, Antwerp, Belgium; 6 Service de Néphrologie, Cliniques Universitaires Saint-Luc, Université Catholique de Louvain, Brussels, Belgium; 7 Department of Gastroenterology and Hepatology, University Hospitals KU Leuven, Leuven, Belgium; 8 Service Gastroentérologie Hépatologie Pédiatrique, Cliniques Universitaires St. Luc, Université Catholique de Louvain, Brussels, Belgium; 9 Department of Hepato-Gastroenterology and Digestive Oncology, CHR Citadelle, Liège, Belgium; 10 Division of Cardiovascular Disorders, Haemophilia Center, University Hospitals KU Leuven, Leuven, Belgium; 11 Department of Clinical Sciences, Institute of Tropical Medicine Antwerp, Antwerp, Belgium; 12 Barcelona Institute for Global Health (ISGlobal), Hospital Clínic, University of Barcelona, Barcelona, Spain

**Keywords:** Disease elimination, Health policy, Hepatitis C, Treatment, Belgium

## Abstract

**Background:**

Hepatitis C virus is one of the leading causes of chronic liver disease and liver-related deaths worldwide. The estimated prevalence of chronic hepatitis C viral infection among the general Belgian population was 0.57% (*n* = 64,000) in 2015. Although Belgium has had a ‘Hepatitis C Plan’ since 2014, elimination efforts are unclear. This study employs the best available data and modelling estimates to define the burden of hepatitis C viral infection among key subgroups in Belgium, identify information gaps and propose potential approaches to screening, linkage to care and treatment, and cure.

**Methods:**

We examined the peer-reviewed and grey literature since 2012 for data on the prevalence of hepatitis C viral infection in Belgium in key subgroups identified by national experts and in the literature. Ultimately, this research is primarily based on data provided by the key stakeholders themselves due to a lack of reliable data in the literature. Based on this, we modelled the treatment rates required to reach elimination of hepatitis C in several subgroups.

**Results:**

Eleven potential subgroups were identified. There were no data available for two subgroups: generational cohorts and men who have sex with men. In six subgroups, fewer than 3000 people were reported or estimated to have hepatitis C infection. Migrants and people who inject drugs were the most affected subgroups, and children were the least affected subgroup. Only two subgroups are on target to achieve elimination by 2030: patients living with haemophilia and transplant recipients.

**Conclusions:**

Removing Belgian treatment reimbursement restrictions in January 2019 was a big step towards eliminating HCV. In addition, increasing surveillance, including with a national registry, treatment prescription by other health-care providers and availability of treatment in local pharmacies are central to improving the current situation and getting on track to reach the 2030 WHO hepatitis C elimination targets in Belgium.

## Background

Infection with the hepatitis C virus (HCV) remains one of the leading causes of chronic liver disease and liver-related deaths worldwide [1, 2], prompting the World Health Organization (WHO) in 2016 to define targets for eliminating it by 2030 [3, 4]. The estimated prevalence of chronic HCV infection among the general Belgian population was 0.57% in 2015 [5]. However, estimates are based mostly on modelling data, as recent population-based studies are scarce and subject to selection bias [6]. Since 2014, Belgium has had a ‘Hepatitis C Plan’ [7]. The plan was developed in collaboration with healthcare professionals (e.g. gastroenterologists specialized in hepatology, representatives from professional associations of gastroenterologists, doctors associated with AIDS referral centres, and representatives from addiction treatment centres) and with patient associations. The main goals of the plan were: (1) to reduce transmission; (2) to increase the number of HCV-positive patients aware of their diagnosis; and (3) to enhance the patient care pathway and quality of life [7], and were listed up in 23 different action points. Many action points, including a screening policy and the collection of scientific data, have not yet been executed and it is unclear how the action points will be evaluated [8, 9]. Therefore, it has been difficult to create an overview of HCV elimination efforts in Belgium.

A practical, comprehensive approach to national HCV elimination can benefit from population segmentation in order to tailor concentrated elimination efforts to specific population subgroups—a concept known as ‘micro-elimination’ [10, 11]. These population subgroups include children (under the age of 15 years), HCV/HIV-coinfected persons, birth cohorts, haemodialysis (HD) patients, those living with haemophilia, men who have sex with men (MSM), migrants, people with advanced liver disease, people who inject drugs (PWID), prisoners, and transplant recipients (Table 1) [10, 22]. However, accurate, up-to-date prevalence data to inform strategic micro-elimination planning are lacking in Belgium.

**Table 1:** Target population for micro-elimination of HCV in Belgium

SUBGROUP	Estimated number of Hepatitis c infections
Children (under age 15)	Newly diagnosed (and reported) HCV cases in 2015: 19 cases aged under 15 years of age [12]
Coinfected with HIV	353 (calculated, 2.4% of HIV-positive population) [13, 14]
Generational cohorts with high prevalence (born 1945–1965)	No data
Patients on HD	297 (4% of patients on HD in Belgium) [15]
Haemophilia patients	No national data; 11 patients remaining at the University Hospital Leuven
MSM	No national data: 19 out of 1055 (1.8%) in 2017 at ITM Antwerp
Migrants from high-prevalence countries	18,607 estimated (range 9729-32,764) [16]
Patients with advanced liver disease	21,875 [17, 18]
PWID	2970 calculated used benchmark multiplier methodology [19]
Prisoners	1777 calculated (15.1% HCV-infected of 11,769 prisoners) [20, 21]
Transplant recipients	146 (12.6%) out of 1156 liver transplants between 2008 and 2012 [6]; 11 at one site [6]

Abbreviations: *HCV* hepatitis C virus; *HD* haemodialysis; *MSM* men who have sex with men; *ITM* Institute of Tropical Medicine; *PWID* people who inject drugs

With direct-acting antiviral (DAA) therapy and its ≥95% cure rate, HCV elimination is achievable [23, 24]. Belgium based its 2015 guidelines for prescribing DAA therapy on the fibrosis stage, restricting it to patients with severe fibrosis or cirrhosis (F3–F4). Since January 2017 DAA therapy was reimbursable not only for patients starting treatment from the F2 fibrosis stage, but for all patients with risk factors or comorbidities such as hepatitis B virus (HBV)- or HIV-coinfection, haemophilia, and severe extrahepatic manifestations, as well as patients on dialysis or those on a transplant waiting list [9, 23–26]. Since the latest update in January 2019 HCV treatment is reimbursed for every patient regardless of the degree of fibrosis [27]. Currently, DAAs can be prescribed and initiated only by a gastroenterologist and are available only in a hospital pharmacy [26].

In this paper, we attempt to employ the best available data to define the burden of HCV infection among key subgroups in Belgium, identify information gaps among these subgroups, and propose potential approaches for screening, prevention, linkage to care, treatment and, ultimately, cure. To assess the feasibility of reaching the WHO elimination goals, we further present mathematical modelling results.

## Methods

A constrained optimization modelling approach was applied for developing the model in Excel. This modeling approach is commonly applied in healthcare for purposes such as capacity management, clinical decision making, and optimal allocation of resources [28]. The model was designed to address two objectives. The first objective of this model is to demonstrate the number of HCV patient needed to treat per year in order to reach the WHO targets for HCV elimination given constraints such as the number of known HCV patients, the country specific annual incidence rate of HCV and the number of years until the year 2030 is reached.

The first objective function is:

$$ \mathrm{X}\ast \frac{\mathrm{Y}{\left(1+\mathrm{Y}\right)}^{\mathrm{t}}}{{\left(1+\mathrm{Y}\right)}^{\mathrm{t}}-1}=\mathrm{N} $$

N = number of patients needed to treat per year to reach the WHO HCV elimination goal by 2030.

X: number of HCV patient in a given year.

Y: country specific HCV incidence rate.

t: number of years.

The second objective of the model is to demonstrate the year in which HCV elimination will be reached with the current treatment rate or alternative treatment rates.

The second objective function is:

$$ \sum \limits_{\mathrm{i}=0}^{\mathrm{T}}\frac{{\mathrm{C}}_{\mathrm{i}}}{{\left(1+\mathrm{y}\right)}^{\mathrm{i}}}=0 $$

T = the year HCV elimination will be reached.

i = number of years.

C: number of HCV patient (= number of eligible patients – number of patients treated).

y: country specific HCV incidence rate.

The estimates quantifying the subpopulations used in the model to project were:

- the number of patients who would receive treatment and the number of patients who would be left untreated by the year 2030 based on the current (3%) versus alternative (needed to meet the WHO targets) rate (8%) of treatment per year.
- year in which HCV elimination will be reached in that group with the current treatment rates or alternative treatment rate;

The input parameters used were as follows:

- estimated number of HCV patients eligible for treatment
- incidence rate of HCV infection
- rate of reinfection
- annual treatment rate

We searched the peer-reviewed (PubMed/Medline) and grey literature (Google Scholar) between January 2012 and August 2018 for data on HCV prevalence in Belgium in key subgroups. The limits are set so that the emphasis is on HCV elimination in the DAA era. Since 2012, DAA treatment has been available in Belgium, initially in a study context. Keywords included the subgroups affected by HCV identified by Lazarus et al. (Table 1), variations in HCV genotypes and differences between the subgroups in Belgium [10, 11, 22]: hepatitis c virus (HCV), prevalence, health policy, Belgium, children, people who inject drugs (PWID), migrants, HIV, men who have sex with men (MSM), prisoners, liver transplant, haemodialysis, haemophilia, generational cohort and advanced liver disease. Little to no data have been published on the prevalence of HCV in the key subgroups listened in Table 1. Therefore, this research is primarily based on data provided by the key stakeholders themselves listed as co-authors in this article, and not on a literature review. Ten national experts were contacted to provide (unpublished) data mainly on the prevalence of HCV in the aforementioned subgroups. All experts graduated as medical doctors in different specialisations with a common interest in HCV. We reported data for notified cases and estimates, as well as fibrosis stage, where available. These data were supplemented with original data from the 2017 Hep-CORE questionnaire [29].

## Results

### Children (under age 15)

There are no peer-reviewed sources available on the current prevalence and incidence of HCV infection in children in Belgium. The 2016 annual report from the Belgian Public Health Research Institute pooled data of a sentinel laboratory network in Belgium and reported that fewer than 15 children were genotyped for HCV in 2015 (Table 2) [5]. Furthermore, based on pooled data from all of the health insurance distributors in Belgium, even fewer children received HCV serology testing in 2016 [30]. Alternatively, an attempt was made to calculate the rate of mother-to-child transmission. Assuming an incidence of 0.57% of HCV in adult patients in Belgium and a rate of 100,000 births each year, 500 women infected with HCV give birth with a 5.0% risk of perinatal transmission [31]. This leads to an estimated 25 infected infants per year.

**Table 2:** Number of children infected with HCV, treatment and cure rates in University Clinics Saint-Luc, Brussels

	Age	Patients (total, n)	Treated (n)	SVR reached (n)
< 15 years	3 months	1	0	0
	13 months	1	0	0
	3 years	2	1	1
	4 years	1	1	1
	5 years	3	0	0
	8 years	2	2	2
	13 years	5	5	5
*Subtotal*		*15*	*9*	*9*
> 15–18 years		10	–	–
TOTAL		25	9	9

Abbreviations: *SVR* sustained virological response

### HIV-positive patients

The incidence of HCV in an HIV cohort in Antwerp was estimated to be between 0.24 (2001) and 1.36 (2011) new cases per 100 person-years, with a peak of 2.93 new cases per 100 person-years in 2009 [32, 33]. The prevalence of HCV/HIV coinfection among this cohort of close to 1400 MSM was 10% in 2017. In 2014, Belgium counted 14,719 people living with HIV (PLHIV) of whom 3% would be HCV/HIV co-infected [13].

### Men who have sex with men

Screening for HCV is not yet recommended in the HIV-negative MSM population and no data are available on the prevalence of HCV in this subgroup in Belgium. Routine data from a screening centre for sexually transmitted infections (STIs) in Antwerp, focusing on people belonging to difficult-to-reach, high-risk groups (including MSM, migrants and sex workers) show that in 2017, 19 out of 1055 people tested (1.8%) were HCV-positive.

### Generational cohorts with high prevalence

In 2016, 1106 patients were screened for HCV antibodies (Ab) at the emergency department (ED) of a tertiary hospital in Ghent [34]. The overall prevalence of HCV Ab was 1.9%. There was no significant difference in the prevalence of HCV in the different birth cohorts, and HCV positivity was solely linked to a history of injecting drug use. A second study was performed at the ED of Ziekenhuis Oost-Limburg (ZOL) Genk in 2017 [35]. In total, 2366 patients aged 18–70 years were screened for HCV Ab and HCV ribonucleic acid (RNA). The prevalence was 1.31% for HCV Ab and 0.52% for HCV RNA. Two thirds of the patients were born between 1955 and 1974. HCV Ab positivity was linked to being a PWID, imprisonment, and birth in an high-endemic country with an HCV Ab prevalence > 2.9%.

### Haemophilia patients

The University Hospital Leuven has one of the largest groups of haemophilia patients in Belgium and is on track to reach the WHO elimination goals, with 11 patients still left untreated of the initial 84 HCV RNA-positive persons eligible for DAA treatment (Frederik Nevens, Kathelijne Peerlinck, September 2018, oral communication, unreferenced).

### Haemodialysis patients

In the early 1990s, a high prevalence of HCV infection among patients in HD units and nosocomial infections was documented [36, 37]. Since then, strict hygienic measures have been taken to reduce the risk of HCV transmission through HD [38]. The HCV Ab prevalence decreased from 13.5% (1991) to 6.8% (2000) in the Belgian dialysis cohort (*n* = 1710) in 15 hospitals (*p* < 0.001) [36]. More recently in 2014, an HCV prevalence of 4.1% in Belgium was described in the multinational, multicentre Dialysis Outcomes and Practice Patterns Study (DOPPS) cohort [15]. Treatment is not always provided to HD patients due to multi-morbidity, which carries a higher risk of drug–drug interactions [39–41].

### Migrants from high-prevalence countries

As previously described, one Belgian study at the ED of ZOL, Genk confirmed that HCV Ab prevalence was independently associated with coming from a country with high HCV endemicity [35]. According to a report of the European Centre for Disease Prevention and Control in 2016, 11.4% of the Belgian adult population was born in a country with high HCV Ab endemicity (HCV Ab prevalence > 1%) [42].

### Patients with advanced liver disease

Experts from the Belgian Association for Study of the Liver (BASL) together with the Polaris Institute estimated that, in 2014, there were 64,340 (0.57%) HCV-infected patients, including 13,961 (21.7%) in F3 and 7914 (12.3%) in F4 stages in Belgium [43].

An estimation of the number of treated patients can be made using the ‘Farmanet’ data from the National Institute for Health and Disability Insurance. These provided the defined daily dose (DDD) of all antiviral therapies distributed by pharmacies in Belgium for 2015 and 2016 (Table 3) [44]. Assuming that all Belgian patients with hepatitis are treated with antivirals accessed in Belgium, we can estimate a range based on short and long treatment duration of patients treated with reimbursed DAA therapy in 2015 and 2016 by dividing the DDD through the number of days per treatment. Since treatment duration can extend from 84 days to as many as 168 days, which is likely in patients with cirrhosis [45]. Of 21,875 estimated patients with advanced liver disease, only 2323 were treated with DAA therapy in 2015–2016.

**Table 3:** Provision of defined daily doses of DAA therapy and estimated number of treated patients

	2015		2016		Total	
	DDD (n)	Total (n)	DDD (n)	Total (n)	DDD (n)	Total (n)
SOF	107,075	637–1275	37,075	221–441	144,150	858–1716
SOF/LDV	1036	6–12	21,172	126–252	22,208	132–264
OBV/PTV/r	8988	54–107	19,844	118–236	28,832	172–343
Total	117,099	697–1394	78,091	465–929	195,190	1162-2323

Abbreviations: *DDD* defined daily dose; *SOF* sofosbuvir; *LDV* ledipasvir; *OBV/PTV/r* ombitasvir, paritaprevir and ritonavir

### People who inject drugs

In Belgium, the population size of PWID was estimated using a benchmark multiplier methodology in 2013 [19]. In 2015, there were an estimated 9080 PWID, 47% of whom were engaged in harm reduction efforts, including needle–syringe programs (NSP) and/or opioid substitution therapy (OST) [46]. Overall, 2970 PWID were HCV-infected, with an estimated 160 new HCV infections in 2015.

Currently, there are different screening projects running in Brussels, Antwerp, Liège, and in the province of Limburg. The results of these will need to be combined to provide data on the uptake of screening and treatment, and to study the incidence of HCV after introduction of DAA therapy. In a large, multicentre retrospective analysis in 15 hospitals across Belgium, 35 patients on OST and 18 active PWID were treated between 2013 and 2015 with DAA monotherapy [20]. This means < 1% of HCV-infected PWID were being treated annually.

### Prisoners

There were 11,769 prisoners in 34 Belgian prisons in 2014 [47]. The HCV Ab positivity rate among prisoners in Belgium is estimated to be around 10–15%, according to a retrospective study in one prison in Liège (Mémoire Virginie Minguet 2010–2011; unpublished). In this study, 3710 consecutive prison entrants between 2006 and 2008 were screened for HCV Ab. Of them, 542 (14.6%) tested positive (Table 4). It is difficult to extrapolate these results to other Belgian prisons, since the population can vary from one prison to another.

**Table 4:** Baseline characteristics of HCV Ab-positive prisoners in Liège

Variable		*n* (%)	M ± SD	*p*-value
Age (years)	Total (M ± SD)	542	35.7 ± 7.37	
	Male (M ± SD)	460 (84.6%)	35.9 ± 7.46	0.29
	Female (M ± SD)	84 (15.4%)	35.0 ± 6.85	
Ethnicity	Western Caucasian	425 (78.1%)	–	–
	North African	86 (15.8%)		
	Eastern Caucasian	31 (5.7%)		
	Southern African	2 (0.4%)		
Coinfection HIV	No	515 (94.7%)	–	–
	Yes	29 (5.3%)		
Drug abuse	No	61 (11.5%)	–	–
	Yes	471 (88.5%)		
Reinfection after previous cure in prison (*n* = 15)	No	10 (66.6%)	–	–
	Yes	5 (33.3%)		

### Transplant recipients

Transplanted patients receive regular follow-up care in a tertiary hospital. At the University Hospital Leuven, 40 patients received a liver transplant due to HCV-related hepatocellular carcinoma (HCC) or end-stage liver disease since the beginning of recording in 1989 (Table 5). Of them, only 15 still have HCV infection; seven have multiple comorbidities, seven are living abroad and one is lost to follow up.

**Table 5:** Micro-elimination of HCV in transplant recipients in Belgium

	Transplant UZ Leuven (n)
Total liver transplants	1360
Liver transplantation due to HCV:	169
HCC	40
Decompensated cirrhosis	129
Patients still alive	66
Relapse of HCV after transplant	65
Treated for HCV relapse	55
IFN	21
DAA	34
Cured of HCV relapse	50
IFN	16
DAA	34
Patients with HCV no longer in follow-up	15
Living abroad	7
No treatment (comorbidity)	7
Loss to follow-up	1

Abbreviations: *HCV* hepatitis C virus; *HCC* hepatocellular carcinoma; *IFN* interferon; *DAA* direct-acting antiviral (drug)

### Mathematical model

According to the mathematical model that was employed in this study, none of the subgroups added to the model would currently achieve HCV elimination by 2030. With the current treatment rate of 3%, the first subgroups, namely migrants, HD patients and prisoners, would reach HCV elimination by 2056 at the earliest (Table 6). If Belgium is to meet the WHO targets, the average percentage of patients to be treated per year out of the total pool of patients who require treatment needs to increase to 8% (Table 7).

**Table 6:** Mathematical modelling results based on current Belgian situation

	RESULTS BASED ON CURRENT SITUATION					
POPULATION Subgroup	Number of patients to treat at 80% of the estimated patient pool in 2030	Number of patients treated by 2030	Number of patients left untreated by 2030	% of patients not treated	Average number of patients treated per year between 2018 and 2030	The year elimination reached
PLHIV^a^	282	92	190	67%	7	2057
HD patients^a^	238	78	160	67%	6	2056
Migrants ^b^	14,969	4882	10,087	67%	375	2056
Patients with advanced liver disease^b^	17,500	5722	11,778	67%	440	2057
PWID^b^	3656	996	2660	73%	77	2314
Prisoners^b^	1422	465	957	67%	36	2056

^a^ Reported ^b^Estimated

Abbreviations: *PLHIV* people living with HIV; *HD* haemodialysis; *PWID* people who inject drugs

**Table 7:** Mathematical modelling results-based simulation to reach target

	RESULTS-BASED SIMULATION TO REACH TARGET
SUBGROUP	Number of patients needed to treat per year between 2018 and 2030 to reach elimination target^a^ by 2030
PLHIV	21
HD patients	18
Migrants	1145
Patients with advanced liver disease	1346
PWID	281
Prisoners	109
Total	2643

^a^Treatment target was assumed at treating 80% of the HCV-infected population

Abbreviations: *PLHIV* people living with HIV; *HD* haemodialysis; *PWID* people who inject drugs

## Discussion

The targets set out by WHO for the elimination of viral hepatitis by 2030 are highly ambitious and whether it is possible for each Member State to reach these remains unclear. In Belgium, this study found that more effective efforts are required. BASL recommends targeted screening for the aforementioned key populations [17, 48]. Nevertheless, this is not yet being done in Belgium, notwithstanding the serious health and economic burden associated with chronic HCV infection [49]. Despite the efforts that were made to prepare the Belgian Hepatitis C plan, HCV elimination among these groups has not been documented to date, and there is no surveillance available to monitor the elimination of HCV infection [7, 8]. Additionally, DAA treatment has been available since 2015 and, although it is only reimbursed for every patient regardless of the degree of fibrosis since January 2019 [27]. The HCV prevalence in the general Belgian population is low, although based on the limited data available, it seems slightly higher in several subgroups. Therefore, it may be more feasible to approach these smaller populations with a micro-elimination approach. It is less daunting, less complex and less costly than full-scale country-level initiatives [10]. Further, if this micro-elimination strategy initially focuses on patients already in the health system, prevention, screening and treatment interventions can often be provided more easily. Stakeholders who are not traditionally involved with viral hepatitis could be more interested in engaging due to this efficient way of increasing diagnosis and treatment in selected populations.

### Addressing micro-elimination in subpopulations

#### Children (under the age of 15)

Worldwide, 5 million children under the age of 15 are chronically infected with HCV [50]. A higher infection prevalence (1.8–5%) is reported in low- and middle-income countries and a lower prevalence is reported (0.05–0.36%) in high-income countries [50, 51]. The primary source of infection among children is mother-to-child transmission [52]. Testing for HCV RNA in children born to mothers with chronic HCV infection is reimbursed in Belgium. Infected infants often experience spontaneous clearance, RNA tests are expensive and there is no safe HCV treatment yet available for children under the age of 3 years. Therefore, experts recommend that children be tested at the age of 18 months or older [53]. There are no recommendations in Belgium yet on the use of DAA therapy in children, nor is there information on the follow up of children with chronic HCV infection.

#### Patients who are already in the health-care system

##### Patients with advanced liver disease

In 2017, 46% of all European countries still had a reimbursement restriction requiring a fibrosis stage of ≥F2 [23, 24]. Recently, a consensus statement redefined these fibrosis stages using different diagnostic tools [18]. Treating patients with more severe liver disease (F2–F4) is effective in reducing all-cause and liver-related morbidity and mortality; however, it will have less impact on transmission, and treating only these patients will be insufficient to reach the WHO targets [54]. Further, we still need to define when a patient will not benefit from DAA therapy and requires a transplant. Following the EASL guidelines of 2018, a transplantation before HCV treatment is recommended if the patient has decompensated cirrhosis and a MELD score ≥ 18–20, because the probability of significant improvement in liver function and delisting is low [55–59].

##### Haemophilia patients

The prevalence of HCV infection in haemophilia patients varies by country, reaching as high as 100% in some reports [60]. Up to 20–30% of these patients develop end-stage liver disease or HCC, which has become the most prevalent cancer in this subgroup [61]. Since January 2017, patients without any ongoing high-risk behaviour for acquiring HCV could be treated with a minimal risk of reinfection in Belgium. In Ireland, more than 90% of all haemophilia patients with HCV infection were cured with a DAA regimen. As a result, HCV infection was eradicated in this Irish subgroup in 2016 [62]. At the University Hospital Leuven, elimination has almost been reached, with just 11 patients untreated. However, we should have a monitoring tool to generate these data nationwide.

##### Haemodialysis patients

Overall, the HCV Ab prevalence has declined in HD patients, according to the DOPPS cohort [15]. Nevertheless, between 2012 and 2014, only 1.7% of DOPPS patients who were HCV Ab-positive were treated at any point during study follow up, and 35% of facilities used isolation stations for HCV-infected patients, a significant, yet debated, logistical burden [39]. Importantly, HCV Ab-positive patients had higher adjusted rates of mortality (HR = 1.14, 95% confidence interval [CI]: 1.06–1.23) and time to first hospitalization (HR = 1.10, 95% CI: 1.06–1.15) [40]. Since the introduction of strict hygienic measures, there are only around 297 HCV-infected HD patients remaining in Belgium [15]. Elimination of HCV is feasible in this subgroup as they are under strict follow up, regularly screened for HCV, and have unrestricted access to therapy with DAAs. Furthermore, the availability of a new, well-tolerated pangenotypic treatment regimen will make DAA treatment possible for all HD patients [62]. The elimination of HCV would have the additional advantage of preventing further nosocomial transmission within HD units [63].

##### Transplant recipients

There has been a significant decrease in the prevalence of patients receiving a transplant due to evolving antiviral therapy [64]. The new DAA regimens allow treatment of patients with cirrhosis, with a MELD score of up to 18 [65]. Collection of data is necessary to document this success and there is not yet a centralised database of transplanted patients in Belgium. Currently, Belgium is on track to eliminating HCV in this subgroup by 2030.

##### HIV-positive patients

A systematic review estimated that there were approximately 2,278,400 HIV/HCV-coinfected persons globally, accounting for an overall coinfection prevalence of 6.2% among PLHIV in 2016 [14]. Since 2017, all HIV/HCV-coinfected patients can be treated with DAA therapy in Belgium. This creates an opportunity to eliminate HCV in this well-defined and well-linked-to-care cohort of patients. However, the effect of DAA therapy on the incidence of HCV among PLHIV has not been thoroughly studied. This remains a concern, as reinfection is not uncommon [66]. We can only speculate that reinfection would be increased in the subgroup compared to the general population in Belgium as we lack data on this subject. Systematic testing has proven to be effective in achieving high rates of HCV diagnoses [67]. All PLHIV in Belgium who engage in sexual risk behaviour and visit an outpatient clinic in an AIDS reference centre should routinely be screened for HCV infection at least once a year [68].

#### People potentially at risk due to demographic characteristics

##### Generational cohorts with high prevalence

The Centers for Disease Control and Prevention (CDC) recommended one-time testing for all persons born between 1945 and 1965 in the United States of America (USA) [69]. The CDC estimated that in the USA, 800,000 persons unaware of their HCV infection would be identified and more than 120,000 HCV-related deaths would be averted if these persons were linked to appropriate HCV care [70, 71]. Due to the lack of data it is unclear whether this would be comparable to the Belgian population. A study on birth cohort variation in liver cirrhosis between eight European countries calculated that more than 50% of all patients were born between 1955 and 1974, while approximately 70% were born between 1950 and 1979 [6, 72]. In a recent Belgian study conducted at the ED of ZOL Genk, there was no significant difference in the prevalence of HCV Ab in the different birth cohorts. HCV Ab positivity was linked solely to a history of injecting drug use [35]. Targeting males born between 1955 and 1974 in Belgium could be a possible screening approach, though only if we were not able to focus on screening high-risk groups. In general, there remains a lack of prospective, representative studies powered to analyse the distribution of HCV by birth cohort, while correcting for confounders such as risk behaviour.

##### Migrants from high-prevalence countries

In 2016, Belgium received 18,710 asylum requests [16]. Of these, 2766 people from Syria requested asylum and 724 people from Russia, both countries with chronic HCV infection rates of > 2.9%. Approximately 100 of those migrants would be chronically infected with HCV. The average HCV Ab prevalence for migrants in Belgium was estimated at 1.8%, meaning 18,607 (9729–32,764) migrants would be HCV Ab-positive. It is difficult to state whether universal screening of migrants for HCV would be cost effective [73]. Models have shown that universal screening would not be cost effective, upon which experts have recommended that migrant populations be screened only if the HCV prevalence is > 2.9% in the country of origin [74]. Currently, there are no real-life data available on the prevalence of HCV infection in, or linkage to care or treatment of HCV-infected, migrants in Belgium.

#### People with high-risk behaviour

##### People who inject drugs

In most high-income countries, PWID are at the heart of the ongoing HCV epidemic [75]. To avert future liver disease and new infections, prevention of HCV transmission among PWID is crucial [76]. The Belgian Hepatitis C Plan, BASL and European Association for Study of the Liver (EASL) guidelines recommend screening PWID for HCV infection, yet there is no reimbursement for repeated HCV RNA testing [7, 25, 26, 45]. The European Monitoring Centre for Drugs and Drug Addiction has stated that combined OST and high-coverage NSP can reduce HCV incidence by up to 80% [77–81]. The HCV incidence remains high in many settings, particularly in the first several years after a person starts injecting drugs [82–85]. Diagnosis rates have increased through awareness campaigns, screening programmes providing free and anonymous testing to PWID, and training of general practitioners to identify risk factors. Furthermore, moving testing out of hospitals and into addiction clinics, harm reduction programmes and other services targeting PWID, as well as referring them directly to specialists from these settings, have proven to be the best paths to reaching this group effectively [46, 80, 83]. A similar project on outreach and treatment under one roof is currently conducted in Antwerp and Limburg. In a case management study in Limburg, results revealed that some target groups were not reached, such as young opiate injectors who had not yet contacted centres such as CAD Limburg or Free Clinic Antwerp, and injectors using injectable drugs other than heroin [86]. At present, a targeted screening strategy is being conducted only with the help of external funding through projects. Additionally, if DAAs could be prescribed by general practitioners and were available in local pharmacies, the threshold for linkage to care would be lower. Improving harm reduction and scaling up treatment uptake to ≥10% annually is an absolute necessity to reach the targets of WHO [3, 21].

##### Prisoners

In prisons, the estimated HCV Ab prevalence is 15.4% in Western Europe, and 20.7% in Eastern Europe [87]. However, these estimates are based on few studies, and could even be an underestimation as was shown in Ukraine [88]. Awareness campaigns and screenings have shown an improvement in the diagnosis rate within prisons. Interventions in France, Italy and Scotland have been successful when prisoners could be treated directly in the prisons [67]. A national plan could be easily implemented by the Ministry of Health in conjunction with the Ministry of Justice. This plan should not only include screening of prisoners but also training of health-care providers in prisons and defining the methodology to access treatment. All prisoners in Belgium have the right to get treated for an HCV infection. However, there is no universal screening procedure yet. For further testing (FibroScan®), prisoners need to be transferred to one of the two prisons with a hospitalisation unit (Liège and Bruges). Low treatment uptake in prison could be explained by difficulties in transfer to the referral centres but also by different reasons (Mémoire Virginie Minguet 2010–2011; unpublished) such as a short length of incarceration and reluctance to take treatment. As all health-care costs are provided by the Federal Department of Justice, large-scale screening and treatment is not a priority. Due to the lack of data, it is not clear how much overlap there is between prisoners and PWID or also the migrant population. This is important to know given that where there is overlap and we can treat all infected prisoners then we can also reduce the number of infected individuals in the overlapping risk groups.

##### Men who have sex with men

Studies from large cities suggest that HIV-negative MSM are also at risk for HCV infection, especially those on pre-exposure prophylaxis (PrEP) [89–92]. PrEP is effective in reducing the incidence of HIV among people who practise high-risk sexual behaviour, though it does not protect them against other STIs such as HCV. A generalised screening through dried blood spots in Barcelona did not detect any incident HCV in this subgroup. In contrast, high-risk behaviour and the incidence of other STIs suggested that periodic screening could be necessary in MSM [93]. Screening the specific subgroup with high-risk behaviour on PrEP is needed and is currently part of the reimbursement criteria for PrEP medication.

### Mathematical model

According to the mathematical model that was used in this study, none of the subgroups added to the model would currently achieve HCV elimination by 2030. The focus of the model is ‘treatment’ as since January 2019 all infected patients in Belgium can be treated regardless of fibrosis stage. Nevertheless, we must keep in mind that HCV elimination goals also relate to the diagnosis of the undiagnosed. Several studies on this subject are currently ongoing in Belgium. Therefore, this is not discussed in this article.

### Limitations

This study is based on grey literature provided by national experts and a scientific literature review. Given the lack of available data, it is not a comprehensive picture of the current situation in Belgium. The population groups mentioned in the study are not uniform. For example, the age of haemophilia patients can greatly influence HCV rates. It is not clear how much overlap there is between the different subgroups described in this study. In the event of an overlap, the treatment of one group may contribute to a reduction in infection in another subgroup.

The model developed for this study serves its purpose: to broadly determine if Belgium is on track to eliminating HCV in specific affected populations. This differs from dynamic transmission models by not taking into account the effects of treatment outcomes with anti-HCV medications or HCV death rates, among other factors. The model assumes an incidence of 2 per 100 person-years and reinfection rate of 2.3% in the PWID population, while due to lack of data, it assumes no additional HCV infections in the other subgroups [94, 95]. Nevertheless, it provides a clear picture of the current situation, which urgently needs to be improved.

## Conclusion

In Belgium, reliable data are scarce on the subgroups investigated in this study. Many are already in specialist care and, as such, present unique and important opportunities to move toward HCV elimination, given that they are not restricted from treatment. Currently, only those living with haemophilia and transplant recipients are on track for reaching HCV elimination by 2030. In contrast, none of the subgroups added to the model would achieve HCV elimination by 2030 at the current treatment rate. To eliminate HCV, more people must be tested and linked to care, and the average percentage of patients to be treated per year out of the total pool of patients to be treated needs to be fixed at a minimum of 8% of the current patient pool. Removing HCV treatment reimbursement restrictions in January 2019 was a big step forward. In addition, creating a centralised HCV database and constructing a national continuum-of-care figure for each group would be important contributions to improving the current situation. Another challenge is access to DAA therapy. At present, it can be prescribed and initiated only by a gastroenterologist and is available only in a hospital pharmacy. If in the future DAA therapy could be prescribed by other health-care professionals and available in local pharmacies as well, treatment access would improve and patients would be able to receive their medication more conveniently. Ultimately, elimination will be challenging without a vaccine, and therefore adequate prevention measures, including harm reduction for PWID, must be accessible through the country.

## Data Availability

Data sharing is not applicable to this article as no datasets were generated or analyzed during the current study.

## Abbreviations

BASL: Belgian Association for Study of the Liver
CDC: Centers for Disease Control and Prevention
DAA: Direct acting antiviral
DDD: Defined daily dose
DOPPS: Dialysis Outcomes and Practice Patterns Study
EASL: European Association for Study of the Liver
ED: Emergency department
HBV: Hepatitis B virus
HCV Ab: Hepatitis C virus antibodies
HCV: Hepatitis C virus
HD: Haemodialysis
MSM: Men who have sex with men
NSP: Needle syringe program
OST: Opioid substitution therapy
PLHIV: People living with HIV
PrEP: Pre-exposure prophylaxis
PWID: People who inject drugs
RNA: Ribonucleic acid
STI: Sexually transmitted infection
SVR: Sustained virological response;
WHO: World Health Organization
ZOL: Ziekenhuis Oost-Limburg

## References

[1] Polaris Observatory HCV Collaborators. Global prevalence and genotype distribution of hepatitis C virus infection in 2015: a modelling study. Lancet Gastroenterol Hepatol 2017;2:161–176. 10.1016/S2468-1253(16)30181-9 28404132

[2] GBD 2013 Mortality and Causes of Death Collaborators (2015). Global, regional, and national age–sex specific all-cause and cause-specific mortality for 240 causes of death, 1990–2013: a systematic analysis for the Global Burden of Disease Study 2013. Lancet.

[3] World Health Organization (2017). Global hepatitis report, 2017.

[4] WHO (2018). Progress report on access to hepatitis C treatment: focus on overcoming barriers in low- and middle-income countries.

[5] Muyldermans G, Van Gucht S, Van Baelen L. Jaarrapport 2016: hepatitis C virus; Wetenschappelijk Instituut Volksgezondheid - Institution Scientifique de santé Publique (WIV-ISP). https://nrchm.wiv-isp.be/nl/ref_centra_labo/hepatitis/Rapporten/Rapport%20HCV%202016.pdf. Accessed 6 Dec, 2018.

[6] Van Damme P, Laleman W, Stärkel P (2014). Hepatitis C epidemiology in Belgium. Acta Gastroenterol Belg.

[7] Federale Overheidsdienst Volksgezondheid (2014). Veiligheid van de voedselketen en Leefmilieu: Protocolakkoord 'HCV-plan'. Belgisch staatsblad- Moniteur Belge,. In. Brussels.

[8] Mulkay JP: National Hepatitis Plan Belgium. 2017 Viral hepatitis Prevention Board country meeting Belgium, Brussels. http://www.vhpb.org/files/html/Meetings_and_publications/Presentations/BRUS81.pdf. Accessed 5 Jan, 2018.

[9] De Raedt L. Introduction to the Belgian Health System. 2017 Viral Hepatitis Prevention Board country meeting Belgium. http://www.vhpb.org/files/html/Meetings_and_publications/Presentations/BRUS21.pdf. Accessed 6 Dec, 2018.

[10] Lazarus JV, Wiktor S, Colombo M, Thursz M, Foundation EIL (2017). Micro-elimination – a path to global elimination of hepatitis C. J Hepatol.

[11] Lazarus JV, Safreed-Harmon K, Thursz MR (2018). The micro-elimination approach to eliminating hepatitis C: strategic and operational considerations. Semin Liver Dis.

[12] Control ECfDPa. Surveillance Atlas of Infectious Diseases. 2018; https://ecdc.europa.eu/en/surveillance-atlas-infectious-diseases, 2018.

[13] Sasse A et al. Epidemiologie van AIDS en HIV Infectie in België 2014. Wetenschappelijk Instituut Volksgezondheid (WIV-ISP), https://epidemio.wiv-isp.be/ID/reports/Jaarrapport_HIV-AIDS_2014.pdf/. Accessed 1 Jan, 2018.

[14] Platt L, Easterbrook P, Gower E (2016). Prevalence and burden of HCV co-infection in people living with HIV: a global systematic review and meta-analysis. Lancet Infect Dis.

[15] Bieber B, Goodkin DA, Nwankwo N (2015). Hepatitis C prevalence and clinical outcomes in the Dialysis Outcomes and Practice Patterns study. Nephrol Dial Transplant.

[16] Office of the Commissioner General for Refugees and Stateless persons (CGRS) Belgium: Year report 2016. https://www.cgvs.be/nl/cijfers?field_cijfers_month_year_value%5Bvalue%5D%5Byear%5D=2016. Accessed 12 Dec, 2018.

[17] Robaeys G, Van Vlierberghe H, Mathei C, Van Ranst M, Bruckers L, Buntinx F (2006). Similar compliance and effect of treatment in chronic hepatitis C resulting from intravenous drug use in comparison with other infection causes. Gastroenterol Hepatol.

[18] Mauss S, Pol S, Buti M (2017). Late presentation of chronic viral hepatitis for medical care: a consensus definition. BMC Med.

[19] Bollaerts K, Aerts M, Sasse A (2013). Improved benchmark-multiplier method to estimate the prevalence of ever-injecting drug use in Belgium, 2000-10. Arch Public Health.

[20] Bielen R, Moreno C, Van Vlierberghe H (2017). Belgian experience with direct acting antivirals in people who inject drugs. Drug Alcohol Depend.

[21] Matheï CBS, Brixko C, Blach S, Mulkay JP, Razavi H, Robaeys G (2015). The modeled impact of improved Hepatitis C Virus (HCV) treatment strategies on HCV prevalence among people who inject drugs (PWIDs) in Belgium. Abstract presented at.

[22] Lazarus J, Pericàs J, Colombo M, Ninburg M, Wiktor S, Thursz M (2018). Viral hepatitis: “E” is for equitable elimination. J Hepatol.

[23] Marshall AD, Pawlotsky JM, Lazarus JV, Aghemo A, Dore GJ, Grebely J (2018). The removal of DAA restrictions in Europe – one step closer to eliminating HCV as a major public health threat. J Hepatol.

[24] Marshall AD, Cunningham EB, Nielsen S (2018). Restrictions for reimbursement of interferon-free direct-acting antiviral drugs for HCV infection in Europe. Lancet Gastroenterol Hepatol.

[25] Belgian Association for Study of the Liver [website] (2018). Hepatitis C: cut-off’s elastography and biological testing for METAVIR F3-F4 and treatment options in BELGIUM: update 01/06/2018.

[26] Antivirale geneesmiddelen tegen hepatitis C: vergoedingsvoorwaarden vanaf 1 januari 2017 - RIZIV. 2018. http://www.riziv.fgov.be/nl/themas/kost-terugbetaling/door-ziekenfonds/geneesmiddel-gezondheidsproduct/terugbetalen/specialiteiten/wijzigingen/Paginas/antiretrovirale_hepatitisc_terugbetalingsvoorwaarden_20170101.aspx#.Ww0_40iFNPY. Accessed 29 May, 2018.

[27] Antivirale geneesmiddelen tegen hepatitis C: vergoedingsvoorwaarden vanaf 1 januari 2019 - RIZIV. 2019. https://www.inami.fgov.be/nl/themas/kost-terugbetaling/door-ziekenfonds/geneesmiddel-gezondheidsproduct/terugbetalen/specialiteiten/wijzigingen/Paginas/antivirale-hepatitisc-terugbetalingsvoorwaarden_20190101.aspx. Accessed 15 February, 2019.

[28] Crown W, Buyukkaramikli N, Thokala P (2017). Constrained optimization methods in health services research-an introduction: report 1 of the ISPOR optimization methods emerging good practices task force. Value Health.

[29] Lazarus JV, Stumo SR, Harris M (2018). Hep-CORE: a cross-sectional study of the viral hepatitis policy environment reported by patient groups in 25 European countries in 2016 and 2017. J Intern AIDS Soc.

[30] Roberts EA, Yeung L (2002). Maternal-infant transmission of hepatitis C virus infection. Hepatology..

[31] Tovo PA, Calitri C, Scolfaro C, Gabiano C, Garazzino S (2016). Vertically acquired hepatitis C virus infection: correlates of transmission and disease progression. World J Gastroenterol.

[32] Apers L, Koole O, Bottieau E (2013). Incidence of HCV and sexually transmitted diseases among HIV positive MSM in Antwerp, Belgium, 2001-2011. Acta Clin Belg.

[33] Bottieau E, Apers L, Van Esbroeck M, Vandenbruaene M, Florence E (2010). Hepatitis C virus infection in HIV-infected men who have sex with men: sustained rising incidence in Antwerp, Belgium, 2001-2009. Euro Surveill.

[34] Botterman R, Glorieus E, Lefere S, et al. Do or don’t: HCV screening in the Belgian Baby Boom Cohort. In: BWGE2017; 2017; Antwerp. https://bwge2017.sched.com/event/9DfC/do-or-dont-hcv-screening-in-the-belgian-baby-boom-cohort, accessed 6 Dec, 2018.

[35] Bielen R, Kremer C, Koc Ö (2018). Screening for hepatitis C at the emergency department: should baby boomers also be screened in Belgium?. Liver Int.

[36] Jadoul M, Poignet JL, Geddes C (2004). The changing epidemiology of hepatitis C virus (HCV) infection in haemodialysis: European multicentre study. Nephrol Dial Transplant.

[37] Stuyver L, Claeys H, Wyseur A (1996). Hepatitis C virus in a hemodialysis unit: molecular evidence for nosocomial transmission. Kidney Int.

[38] Wheeler D, Winkelmayer WC (2008). The Kidney Disease: Improving Global Outcomes (KDIGO) Clinical practice guidelines for the prevention, diagnosis, evaluation, and treatment of hepatitis C in chronic kidney disease. Kidney Int Suppl.

[39] Goodkin DA, Bieber B, Gillespie B, Robinson BM, Jadoul M (2013). Hepatitis C infection is very rarely treated among hemodialysis patients. Am J Nephrol.

[40] Goodkin DA, Bieber B, Jadoul M, Martin P, Kanda E, Pisoni RL (2017). Mortality, hospitalization, and quality of life among patients with hepatitis C infection on hemodialysis. Clin J Am Soc Nephrol.

[41] Jadoul M, Berenguer MC, Doss W (2018). Executive summary of the 2018 KDIGO hepatitis C in CKD guideline: welcoming advances in evaluation and management. Kidney Int.

[42] ECDC technical report: Epidemiological assessment of hepatitis B and C among migrants in the EU/EEA. 2016. https://ecdc.europa.eu/en/publications-data/epidemiological-assessment-hepatitis-b-and-c-among-migrants-eueea. Accessed 5 Jan, 2018.

[43] Starkel P, Vandijck D, Laleman W (2014). The disease burden of hepatitis C in Belgium: development of a realistic disease control strategy. Acta Gastroenterol Belg.

[44] WHO Collaborating Center for Drug Statistics Methodology. Definition and general considerations of defined daily dose (DDD). https://www.whocc.no/ddd/definition_and_general_considera/. Accessed 14 Mar, 2018.

[45] EASL (2017). Recommendations on treatment of hepatitis C 2016. J Hepatol.

[46] Mathei C, Bourgeois S, Blach S (2016). Mitigating the burden of hepatitis C virus among people who inject drugs in Belgium. Acta Gastroenterol Belg.

[47] Walmsley R (2015). World prison population list (11th edition). World Prison Brief.

[48] Pawlotsky J-M, Negro F (2018). EASL recommendations on treatment of hepatitis C 2018. J Hepatol.

[49] Razavi H, Waked I, Sarrazin C (2014). The present and future disease burden of hepatitis C virus (HCV) infection with today's treatment paradigm. J Viral Hepat.

[50] Sokal E, Nannini P (2017). Hepatitis C virus in children: the global picture. Arch Dis Child.

[51] El-Shabrawi MH, Kamal NM (2013). Burden of pediatric hepatitis C. World J Gastroenterol.

[52] Pawlowska M, Domagalski K, Pniewska A, Smok B, Halota W, Tretyn A (2015). What's new in hepatitis C virus infections in children?. World J Gastroenterol.

[53] Vanhaesebrouck P, Roelens K, De Praeter C, Robberecht E, Goossens L, Leroux-Roels G (2006). Aanpak bij perinatale hepatitis C-virusinfectie. Tijdschrift voor Geneeskunde.

[54] European Union HCV Collaborators (2017). Hepatitis C virus prevalence and level ofintervention required to achieve the WHO targets for elimination in the European Union by 2030: a modelling study. Lancet Gastroenterol Hepatol.

[55] Pascasio JM, Vinaixa C, Ferrer MT (2017). Clinical outcomes of patients undergoing antiviral therapy while awaiting liver transplantation. J Hepatol.

[56] Chhatwal J, Samur S, Kues B (2017). Optimal timing of hepatitis C treatment for patients on the liver transplant waiting list. Hepatology..

[57] Belli LS, Berenguer M, Cortesi PA (2016). Delisting of liver transplant candidates with chronic hepatitis C after viral eradication: a European study. J Hepatol.

[58] Manns M, Samuel D, Gane EJ (2016). Ledipasvir and sofosbuvir plus ribavirin in patients with genotype 1 or 4 hepatitis C virus infection and advanced liver disease: a multicentre, open-label, randomised, phase 2 trial. Lancet Infect Dis.

[59] Charlton M, Everson GT, Flamm SL (2015). Ledipasvir and sofosbuvir plus ribavirin for treatment of HCV infection in patients with advanced liver disease. Gastroenterology..

[60] Franchini M, Tagliaferri A, Rossetti G (2001). The natural history of hepatitis C virus infection in hemophiliacs. Hematology..

[61] Fransen van de Putte DE, Makris M, Fischer K, et al. long-term follow-up of hepatitis C infection in a large cohort of patients with inherited bleeding disorders. J Hepatol 2014;60(1):39–45. 10.1016/j.jhep.2013.08.010 23978717

[62] Irish Haemophilia Society: treatment for hepatitis c; press release 2016. https://haemophilia.ie/hiv-hepatitis-c/hepatitis-c-2/treatment-for-hepatitis-c/. Accessed 5 Jan, 2018.

[63] Jadoul M (1996). Transmission routes of HCV infection in dialysis. Nephrol Dial Transplant.

[64] Goldberg D, Ditah IC, Saeian K (2017). Changes in the prevalence of hepatitis C virus infection, nonalcoholic steatohepatitis, and alcoholic liver disease among patients with cirrhosis or liver failure on the waitlist for liver transplantation. Gastroenterology..

[65] Fernandez Carrillo C, Lens S, Llop E (2017). Treatment of hepatitis C virus infection in patients with cirrhosis and predictive value of model for end-stage liver disease: analysis of data from the Hepa-C registry. Hepatology.

[66] Ingiliz P, Martin TC, Rodger A (2017). HCV reinfection incidence and spontaneous clearance rates in HIV-positive men who have sex with men in Western Europe. J Hepatol.

[67] Gore C, Lazarus JV, Baptista Leite R, et al. Road to elimination: barriers and best practices in hepatitis C management: overview of the status of HCV care in Europe and Australia. Boston Consulting Group, 2017. http://image-src.bcg.com/Images/BCG-Road-to-Elimination_tcm104-166034.pdf. Accessed 5 Jan, 2018.

[68] Apers L, Vanden Berghe W, De Wit S (2015). Risk factors for HCV acquisition among HIV-positive MSM in Belgium. J Acquir Immune Defic Syndr.

[69] Smith BD, Morgan RL, Beckett GA (2012). Recommendations for the identification of chronic hepatitis C virus infection among persons born during 1945–1965. MMWR Recomm Rep.

[70] Rein DB, Smith BD, Wittenborn JS (2012). The cost-effectiveness of birth-cohort screening for hepatitis C antibody in U.S. primary care settings. Ann Intern Med.

[71] US Department of Health and Human Services (2011). Combating the silent epidemic of viral hepatitis: action plan for the prevention, care, and treatment of viral hepatitis. HHS.

[72] Trias-Llimos S, Bijlsma MJ, Janssen F (2017). The role of birth cohorts in long-term trends in liver cirrhosis mortality across eight European countries. Addiction.

[73] Falla AM, Ahmad AA, Duffell E, Noori T, Veldhuijzen IK (2018). Estimating the scale of chronic hepatitis C virus infection in the EU/EEA: a focus on migrants from anti-HCV endemic countries. BMC Infect Dis.

[74] Sharma S, Carballo M, Feld J, Janssen H (2015). Immigration and viral hepatitis. J Hepatol.

[75] Grebely J, Bruneau J, Lazarus JV (2017). Research priorities to achieve universal access to hepatitis C prevention, management and direct-acting antiviral treatment among people who inject drugs. Int J Drug Policy..

[76] European Centre for Disease Prevention and Control (ECDC) (2016). Annual epidemiological report 2016 – hepatitis C.

[77] Degenhardt L, Mathers B, Vickerman P, Rhodes T, Latkin C, Hickman M (2010). Prevention of HIV infection for people who inject drugs: why individual, structural, and combination approaches are needed. Lancet..

[78] Hagan H, Pouget ER, Des Jarlais DC (2011). A systematic review and meta-analysis of interventions to prevent hepatitis C virus infection in people who inject drugs. J Infect Dis.

[79] MacArthur GJ, van Velzen E, Palmateer N (2014). Interventions to prevent HIV and hepatitis C in people who inject drugs: a review of reviews to assess evidence of effectiveness. Int J Drug Policy.

[80] Turner KM, Hutchinson S, Vickerman P (2011). The impact of needle and syringe provision and opiate substitution therapy on the incidence of hepatitis C virus in injecting drug users: pooling of UK evidence. Addiction..

[81] Platt L, Minozzi S, Reed J (2018). Needle syringe programmes and opioid substitution therapy for preventing HCV transmission among people who inject drugs: findings from a Cochrane Review and meta-analysis. Addiction.

[82] Hagan H, Pouget ER, Des Jarlais DC, Lelutiu-Weinberger C (2008). Meta-regression of hepatitis C virus infection in relation to time since onset of illicit drug injection: the influence of time and place. Am J Epidemiol.

[83] Morris MD, Shiboski S, Bruneau J (2017). Geographic differences in temporal incidence trends of hepatitis C virus infection among people who inject drugs: the InC3 collaboration. Clin Infect Dis.

[84] Wiessing L, Ferri M, Grady B (2014). Hepatitis C virus infection epidemiology among people who inject drugs in Europe: a systematic review of data for scaling up treatment and prevention. PLoS One.

[85] Roy E, Nonn E, Haley N, Cox J (2007). Hepatitis C meanings and preventive strategies among street-involved young injection drug users in Montréal. Int J Drug Policy.

[86] Bielen R, Verrando R, Penders J, Oris E, Nevens F, Robaeys G (2016). Case management to improve uptake for screening and therapy of hepatitis C viral infection in people who inject drugs. Hepatology..

[87] Dolan K, Wirtz AL, Moazen B (2016). Global burden of HIV, viral hepatitis, and tuberculosis in prisoners and detainees. Lancet..

[88] Altice FL, Azbel L, Stone J (2016). The perfect storm: incarceration and the high-risk environment perpetuating transmission of HIV, hepatitis C virus, and tuberculosis in Eastern Europe and Central Asia. Lancet..

[89] Richardson D, Fisher M, Sabin CA (2008). Sexual transmission of hepatitis C in MSM may not be confined to those with HIV infection. J Infect Dis.

[90] van de Laar TJ, Paxton WA, Zorgdrager F, Cornelissen M, de Vries HJ (2011). Sexual transmission of hepatitis C virus in human immunodeficiency virus-negative men who have sex with men: a series of case reports. Sex Transm Dis.

[91] Volk JE, Marcus JL, Phengrasamy T, Hare CB (2015). Incident hepatitis C virus infections among users of HIV preexposure prophylaxis in a clinical practice setting. Clin Infect Dis.

[92] Hoornenborg E, Achterbergh RCA (2017). Schim Van Der Loeff MF, et al. men who have sex with men starting pre-exposure prophylaxis (PrEP) are at risk of HCV infection: evidence from the Amsterdam PrEP study. AIDS..

[93] Saludes V, Folch C, Morales-Carmona A (2018). Community-based screening of hepatitis C with a one-step RNA detection algorithm from dried-blood spots: analysis of key populations in Barcelona. Spain J Viral Hepat.

[94] Fraser H, Martin NK, Brummer-Korvenkontio H (2018). Model projections on the impact of HCV treatment in the prevention of HCV transmission among people who inject drugs in Europe. J Hepatol.

[95] Dore G, Grebely J, Altice F (2017). Hepatitis C virus reinfection and injecting risk behavior following elbasvir/grazoprevir treatment in participants on opiate agonist therapy: C-EDGE CO-STAR Part B.

